# Improved strategy for isoleucine ^1^H/^13^C methyl labeling in *Pichia pastoris*

**DOI:** 10.1007/s10858-019-00281-1

**Published:** 2019-09-20

**Authors:** Rustam Ali, Lindsay D. Clark, Jacob A. Zahm, Andrew Lemoff, Karthik Ramesh, Daniel M. Rosenbaum, Michael K. Rosen

**Affiliations:** 1 Department of Biophysics, University of Texas Southwestern Medical Center, Dallas, TX 75390 USA; 2 Howard Hughes Medical Institute, University of Texas Southwestern Medical Center, Dallas, TX 75390 USA; 3 Present Address: Department of Cell Biology, Harvard Medical School, 240 Longwood Avenue, Boston, MA 02115 USA; 4 Present Address: Department of Biological Chemistry and Molecular Pharmacology, Harvard Medical School, 130 Longwood Avenue, Boston, MA 02115 USA; 5 Department of Biochemistry, University of Texas Southwestern Medical Center, Dallas, TX 75390 USA

**Keywords:** ^13^C methyl labeling, Methyl TROSY, Pichia pastoris expression, Deuteration, Eukaryotic protein expression

## Abstract

Site specific methyl labeling combined with methyl TROSY offers a powerful NMR approach to study structure and dynamics of proteins and protein complexes of high molecular weight. Robust and cost-effective methods have been developed for site specific protein ^1^H/^13^C methyl labeling in an otherwise deuterated background in bacteria. However, bacterial systems are not suitable for expression and isotope labeling of many eukaryotic and membrane proteins. The yeast *Pichia pastoris* (*P. pastoris*) is a commonly used host for expression of eukaryotic proteins, and site-specific methyl labeling of perdeuterated eukaryotic proteins has recently been achieved with this system. However, the practical utility of methyl labeling and deuteration in *P. pastoris* is limited by high costs. Here, we describe an improved method for ^1^H/^13^C-labeling of the δ-methyl group of isoleucine residues in a perdeuterated background, which reduces the cost by ≥ 50% without compromising the efficiency of isotope enrichment. We have successfully implemented this method to label actin and a G-protein coupled receptor. Our approach will facilitate studies of the structure and dynamics of eukaryotic proteins by NMR spectroscopy.

**Electronic supplementary material:**

The online version of this article (10.1007/s10858-019-00281-1) contains supplementary material, which is available to authorized users.

## Introduction

Methyl groups are excellent NMR spectroscopic probes of protein structure and dynamics. They are major, spatially dispersed constituents of protein cores. Moreover, their favorable NMR spectroscopic properties afford intense and well separated resonances in ^1^H–^13^C correlation spectra. Methyl groups are relaxed by a network of ^1^H–^1^H and ^1^H–^13^C dipolar interactions. In the macromolecular limit, destructive interference between these interactions leads to extensive cancellation of intra-methyl ^1^H–^1^H and ^1^H–^13^C dipolar interactions in HMQC-based experiments (Ollerenshaw et al. 2003). This effect forms the basis of methyl TROSY NMR spectroscopy, which has enabled quantitative analyses of systems as large as 1.1 MDa (Ruschak and Kay 2012).

Selective ^1^H/^13^C labeling of methyl groups in a perdeuterated background (Rosen et al. 1996; Gardner and Kay 1997) is necessary to exploit the advantages of the methyl TROSY effect in proteins. Robust and cost-effective methods have been developed for site specific methyl labeling in *E. coli* expression systems. However, since bacteria lack many post-translational modification machineries and molecular chaperones, many eukaryotic proteins are not properly modified or folded when expressed in *E. coli*, leading to low yields of desired materials. To circumvent these problems, isotopic labeling methods have been developed in a variety of eukaryotic expression systems, including mammalian cells (Werner et al. 2008), insect cells (Nygaard et al. 2013; Kofuku et al. 2012; Opitz et al. 2015; Sitarska et al. 2015), and yeast (Fan et al. 2011). Although substantial progress has been made in all of these expression systems, yeast provides the greatest opportunity for high-level (> 90%) uniform deuteration, due to the ability of yeast to grow in 100% ^2^H_2_O media.

The methylotrophic yeast, *Pichia pastoris*, is a powerful system for expression of many recombinant eukaryotic proteins. This yeast species combines the advantages of easy genetic manipulation, relatively rapid growth in moderately expensive media, and short expression time. Isotopic labeling in *P. pastoris* was first demonstrated by Laroche et al. 25 years ago (Laroche et al. 1994). Subsequently, several protocols were developed for uniform ^13^C/^15^N- and selective amino acid type-labeling in protonated and perdeuterated backgrounds (Chen et al. 2006; Cereghino and Cregg 2000; Morgan et al. 2000). However, site-specific methyl labeling in a perdeuterated background using α-ketoacid precursors had not been reported in *P. pastoris* until recently by ourselves and Suzuki et al. (Clark et al. 2015; Suzuki et al. 2018). Our strategy required two different media prepared in ^2^H_2_O. A 100% ^2^H_2_O ^2^H-glycerol-rich medium was used for initial stages of cell growth. This medium was then exchanged with a 100% ^2^H_2_O glycerol-deficient medium containing ^1^H/^13^C-labeled methyl amino acid precursors, to induce protein overexpression by addition of ^2^H-methanol. This medium exchange was necessary to prevent inhibition of protein expression from the methanol-activated AOX1 (alcohol oxidase 1) promoter in *P. pastoris*, which is inhibited by glycerol. While improving yields, this exchange substantially increased the cost of the method.

Here we describe a new procedure in which glycerol is directly monitored throughout the growth stage in glycerol rich media, and this stage is extended until glycerol is consumed completely. This new strategy does not require exchanging media, cutting costs nearly 33% by reducing the total ^2^H_2_O used. Furthermore, the optimized media use less deuterated glycerol, which reduces costs by an additional ~ 17–20% relative to the earlier procedure. Combining this new strategy with further optimization of growth conditions, we have been able to increase overexpression yields of ^1^H/^13^C isoleucine δ-methyl-labeled, perdeuterated actin by ~ 30% without compromising isotope enrichment efficiency. We have also implemented this strategy successfully to prepare ^1^H/^13^C isoleucine δ-methyl-labeled A_2A_ adenosine receptor, a G-protein coupled receptor (GPCR), with good yield. Our approach should be extendable to other eukaryotic proteins, and will enable analyses of these systems by NMR spectroscopy at reduced cost.

## Materials and methods

### Optimization of growth medium

The compositions of the old BMGH medium (BMGH, buffered minimal glycerol containing histidine) and the optimized new BMGH (BMGH*) for *P. pastoris* are listed in Table 1. The components (histidine, biotin and glycerol/glycerol-d_8_) were dissolved in 100 mM phosphate buffer (pH 6.0) in 99.9% ^2^H_2_O (Cambridge Isotope Laboratories). To this solution 2.68% (26.8 g/L) YNB with ammonium sulphate and without amino acids (Sigma Aldrich) was added. A 10X or 13.4% YNB stock can be prepared by dissolving 134 g of yeast nitrogen base (YNB) with ammonium sulfate and without amino acids in 1000 mL of water and filter sterilize. Addition of YNB reduces the pH of the medium to 5.6 requiring adjustment back to 6.0 by slow addition of 10 M KOH. The medium was filter sterilized using a 0.2 μM membrane.

**Table 1:** Compositional comparison between glycerol rich old medium (BMGH) and new medium (BMGH*)

Composition (per liter)	BMGH*	BMGH
K_2_HPO_4_	2.29	2.29
KH_2_PO_4_	11.80	11.80
YNB* (g)	26.80	13.40
Glycerol (g)	5.0	10.0
Biotin (mg)	0.8	0.4
Histidine (mg)	80.0	40.0

YNB* yeast nitrogenous base without amino acids

### Optimization of cell growth and actin expression conditions

To compare cell growth and protein expression in the old and new procedures, cells in the two media were grown and expressed using identical conditions. Incubation temperature and shaking speed were maintained at 30 °C and 270 rpm, respectively. Plasmids carrying the previously described (His)_6_-Thymosin β4-actin (D287A, V288A, D289A) mutant construct (Zahm et al. 2013) were transformed into electrocompetent GS115 cells by electroporation. Transformed cells were plated on MDH (Minimal Dextrose with Histidine) agar plates and incubated at 30 °C for ~ 36 h. A single colony was used to inoculate 0.25 mL of BMGH* medium in a 15 mL culture tube, which was incubated with shaking overnight (~ 15 h). The entire culture was used to inoculate 2.5 mL of 50% ^2^H_2_O/50% H_2_O BMGH* medium in a 15 mL culture tube. Cells were incubated with shaking until OD_600_ reached 10-15 (~ 24 h). Cells were centrifuged at 3000×*g* and the medium was decanted. Cells were resuspended in 25 mL of 100% ^2^H_2_O BMGH* medium (with glycerol-d_8_) in a 125 mL baffled Erlenmeyer flask and incubated with shaking until OD_600_ ≥ 15. This starter culture was split into two parts to inoculate 500 mL of either 100% ^2^H_2_O BMGH or BMGH* (both with glycerol-d_8_) in 2 L baffled Erlenmeyer flasks. Both cultures were then incubated with shaking. Throughout the growth, 1 mL samples from each flask were removed at different intervals to measure the OD_600_ and glycerol concentration (see below). For the optimized growth using the new method, once glycerol was no longer measurable in the culture (typically OD_600_ ~ 16–18), the cells were starved for an additional 8 h to ensure complete depletion of glycerol from the medium. For cells growing in BMGH medium, when OD_600_ ~ 16–18, the medium was exchanged with glycerol deficient medium, BMH (buffered minimal containing histidine), and cells were starved for 8 h. At this point, α-ketobutyric acid (methyl-^13^C, 99%; 3,3-D_2_, 98%; Cambridge Isotope Laboratories, MA) (100 mg/L) was directly added to both cultures. Protein expression was induced 3 h later in both cultures by addition of 0.5% (v/v) methanol-d_4_ (Cambridge Isotope Laboratories, MA). An additional 0.5% (v/v) methanol-d_4_ was added 24 h later. After a total of 48 h of expression, cells were harvested by centrifugation at 4700×*g* for 45 min and resuspended in lysis buffer containing 50 mM Tris (pH 8.0), 10 mM imidazole, 2 mM β-mercaptoethanol, 300 mM KCl, 0.2 mM ATP, 0.1 mM CaCl_2_, 1 μg/mL leupeptin, 500 ng/mL pepstatin, 1 mM benzamidine, 1 μg/mL antipain, and 1 mM PMSF. Resuspended cells were either further processed or flash frozen and stored at -80 °C.

### Expression of deuterated adenosine A_2A_ receptor in optimized medium

*P. pastoris* cultures expressing wild-type human adenosine A_2A_ receptor, ADORA2A, were grown from frozen cell stocks previously generated and described (Clark et al. 2017). A single colony from a freshly-streaked MDH plate was used to inoculate 0.5 mL of BMGH* medium in a 15 mL culture tube, and was incubated with shaking (270 rpm) at 28 °C. The entire overnight culture was used to inoculate 5 mL of BMGH* medium prepared in 50% ^2^H_2_O/50% H_2_O. The culture was shaken at 270 rpm at 28 °C until OD_600_ ~ 12–15. Cells were collected by centrifugation at 3000×*g* and resuspended in BMGH* medium prepared in 100% ^2^H_2_O (with glycerol-d_8_). This culture was shaken until OD_600_ ~ 15–20 and used in its entirety to inoculate 2 L of identical media and shaken at 270 rpm at 28 °C. After 24 h of growth, cultures were tested periodically for glycerol content. Once the glycerol was no longer measurable in the medium, the culture was starved for an additional 5 h and then 200 mg/L of labeled α-ketobutyric acid (methyl-^13^C, 99%; 3,3-D_2_, 98%; Cambridge Isotope Laboratories) was added (it may also be possible to use 100 mg/L, although we have retained 200 mg/L for historical reasons). After an additional 3 h, expression was induced with 0.5% (v/v) methanol-d_4_, and the temperature was reduced to 20 °C. Approximately 15 min prior to induction, dry theophylline powder was added to a final concentration of 4 mM. Additional d_4_-methanol was added every 12 h to maintain robust expression. Cells were harvested by centrifugation after 36–48 h and stored at − 80 °C until needed.

### Measurement of glycerol consumption

Glycerol concentration in the medium was measured using a glycerol detection kit (Megazyme, product code K-GLCRL). At different intervals during the growth, cells from 1 mL of culture were collected by centrifugation at 4000×*g* for 10 min. Supernatants were transferred to a microcentrifuge tube and stored at − 20 °C until measurement. The samples were diluted (10X or 100X) so that glycerol concentration was in the linear range of the assay. Appropriately diluted samples (50 μL) were added to a premixed buffer solution containing ATP/PEP (phosphoenolpyruvate)/NADH. Glycerol kinase (10 μL from stock, according to the manufacturer instructions) was added to the resulting mixed solution and incubated for 5 min 37 °C to form glycerolphosphate and ADP. This step was followed by addition of 10 μL pyruvate kinase (PK) for 5 min at 37 °C, to convert ADP into ATP and pyruvate. In the final step, lactate dehydrogenase (10 μL from stock) was added and incubated at 37 °C for 5 min to reduce pyruvate to lactate with oxidation of NADH into NAD^+^. The conversion of NADH into NAD^+^ is stoichiometric with glycerol and measured by a decrease in absorbance at 340 nm, which could be quantified by comparison with a standard curve. The lower limit for glycerol detection by this method is 0.34 mg/L (according to the manufacturer).

### Purification of deuterated G-actin and deuterated adenosine A_2A_ receptor

Actin and adenosine A_2A_ receptor were purified as previously described by Clark et al. (2017) (detailed in supplementary materials).

### Intact mass determination by LC ESI Mass spectrometry

Protein samples were analyzed by LC/MS, using a Sciex X500B Q-ToF mass spectrometer coupled to an Agilent 1290 Infinity II HPLC. Samples were injected onto a POROS R1 reverse-phase column (2.1 × 30 mm, 20 µm particle size, 4000 Å pore size), desalted, and the amount of buffer B was manually increased stepwise until the protein eluted off the column. Buffer A contained 0.1% formic acid in water and buffer B contained 0.1% formic acid in acetonitrile. The mobile phase flow rate was 300 μL/min. The mass spectrometer was controlled by Sciex OS v.1.4 using the following settings: Ion source gas 1, 30 psi; ion source gas 2, 30 psi; curtain gas, 35; CAD gas, 7; temperature, 300 ^°^C; spray voltage, 5500 V; declustering potential, 125 V; and collision energy, 10 V. Data was acquired from 400 to 2000 Da with a 0.5 s accumulation time and 4 time bins summed. The acquired mass spectra for the proteins of interest were deconvoluted using BioPharmaView v. 2.1 software (Sciex) in order to obtain the molecular weights. The reconstruction processing was set to 10 iterations with a signal to noise threshold of ≥ 3 and a resolution of 25,000.

### Isoleucine labeling measured by mass spectrometry

Protein gel pieces were reduced and alkylated with DTT (20 mM) and iodoacetamide (27.5 mM). A 0.1 µg/µL solution of trypsin in 50 mM triethylammonium bicarbonate (TEAB) was added to completely cover the gel, allowed to sit on ice, and then 50 µL of 50 mM TEAB was added and the gel pieces were digested overnight. Following solid-phase extraction cleanup with an Oasis MCX µelution plate (Waters), the resulting peptides were reconstituted in 10 μL of 2% (v/v) acetonitrile (ACN) and 0.1% trifluoroacetic acid in water. 2 μL of this were injected onto an Orbitrap Fusion Lumos mass spectrometer (Thermo Electron) coupled to an Ultimate 3000 RSLC-Nano liquid chromatography system (Dionex). Samples were injected onto a 75 μm inner diameter, 75 cm long EasySpray column (Thermo), and eluted with a gradient from 0 to 28% buffer B over 90 min. Buffer A contained 2% (v/v) ACN and 0.1% formic acid in water, and buffer B contained 80% (v/v) ACN, 10% (v/v) trifluoroethanol, and 0.1% formic acid in water. The mass spectrometer operated in positive ion mode with a source voltage of 1.5 kV and an ion transfer tube temperature of 275 °C. MS scans were acquired at 120,000 resolution in the Orbitrap and up to 10 MS/MS spectra were obtained in the ion trap for each full spectrum acquired using higher-energy collisional dissociation (HCD) for ions with charges 2–7. Dynamic exclusion was set for 25 s after an ion was selected for fragmentation.

Raw MS data files were analyzed using Proteome Discoverer v2.2 (Thermo), with peptide identification performed using Sequest HT searching against the *Drosophila melanogaster* protein database from UniProt. Fragment and precursor tolerances of 10 ppm and 0.6 Da were specified, and three missed cleavages were allowed. Carbamidomethylation of Cys was set as a fixed modification and oxidation of Met was set as a variable modification. The false-discovery rate (FDR) cutoff was 1% for all peptides.

Incorporation efficiency of ^13^C at the δ1 and ^2^H at γ1 positions of isoleucine residues (achieved through growth of yeast in protonated media supplemented with [4-^13^C-3,3-^2^H_2_] α-ketobutyric acid), was quantified by identifying four peptide sequences with high signal/noise and a unique isoleucine (z = + 2 or +3) in the tryptic LC–MS data for both labeled and unlabeled (i.e. fully protonated) actin. Intensity in the isotopic distribution dataset for each peptide was normalized with respect to the intensity of the M + 0 peak (i.e. no ^13^C atoms). Isotope incorporation efficiency in isoleucine for each peptide was calculated by using following equation:

((Relative intensity (M + 3) labeled) − (Relative intensity (M + 3) unlabeled))/((Relative intensity (M + 3) labeled) + (Relative intensity (M) labeled)) × 100

The numerator subtracts off the signal from any naturally occurring signal for the M + 3 peak from ^13^C.

### NMR spectroscopy

NMR data were acquired at 25 °C on an Agilent 600 MHz or 800 MHz NMR spectrometer equipped with a 5 mm cryogenically cooled triple- resonance pulsed field gradient (TRPFG) probe. Two-dimensional (2D) ^1^H/^13^C HMQC TROSY (Tugarinov et al. 2003) spectra were collected with spectral widths of 8000 Hz and 2100 Hz and acquisition times of 64 ms and 28 ms in the ^1^H and ^13^C dimensions, respectively. An inter-scan delay of 1.5 s was employed between successive transients. The states-TPPI mode of quadrature detection was employed for frequency discrimination in the indirectly detected dimension.

Data were processed using NMRPipe (Delaglio et al. 1995). Data sets were zero-filled prior to Fourier transformation. The directly and indirectly detected time domain data were processed by applying a 90° phase-shifted squared sine bell or a Gaussian filter.

## Results

### The new method reduces the cost of CH_3_ labeling by more than 50%

**Fig. 1:**
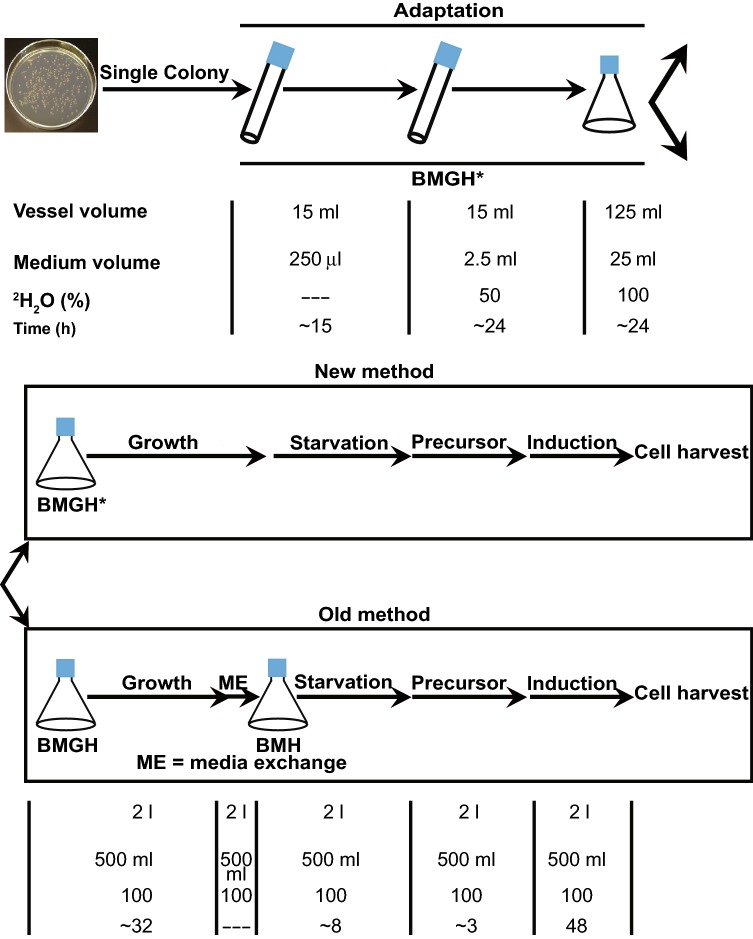
Comparison between the old and new *P. pastoris* expression protocols. The optimized new method does not require exchange of final volume ^2^H_2_O media and uses less glycerol-d_8_, cutting costs by ~ 50% (see text)

In earlier protocols for isoleucine ^13^CH_3_ labeling in *P. pastoris*, a single colony is expanded by successive cycles of growth, collection by centrifugation, and dilution into a larger media volume until the final desired culture size is reached (Fig. 1). These cycles also transition from an H_2_O rich medium to a ^2^H_2_O rich buffered medium containing deuterated-glycerol (1%) as the main carbon source, BMGH (Clark et al. 2015; Suzuki et al. 2018). Since glycerol represses expression from the AOX1 promoter, it must be removed from the final culture before inducing recombinant protein expression. This is typically achieved by exchanging the final culture of deuterated BMGH medium with a similar or equal volume of deuterated, glycerol deficient buffered medium, BMH, through centrifugation and resuspension. Thus, deuterated media are used twice at large volume in the growth. The exchange of BMGH for BMH medium and use of deuterated glycerol in the former account for most of the costs of generating perdeuterated CH_3_ labeled samples. Therefore, an alternative method that minimizes deuterated glycerol and does not involve exchange of the medium is quite desirable.

**Fig. 2:**
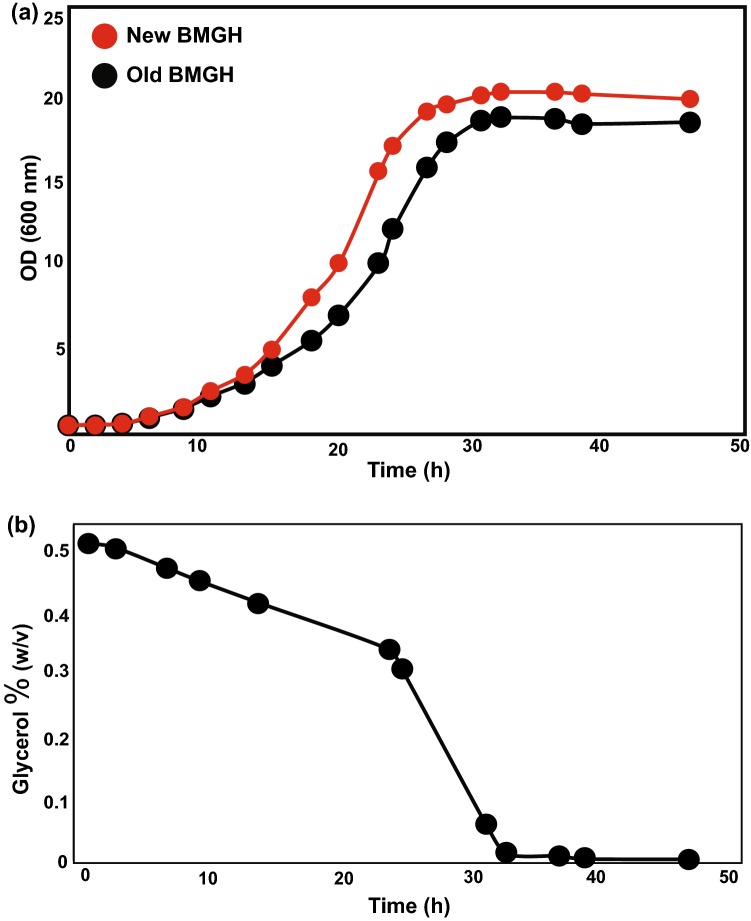
Measurement of glycerol-d_8_ in BMGH* medium and comparison of growth rates of cultures grown in old and new media. (**a**) Cells harboring plasmids expressing actin grow relatively faster and reach a higher density when grown in new optimized medium under otherwise identical conditions. (**b**) Depletion of glycerol-d_8_ as a function of time by *P. pastoris* culture harboring an expression plasmid for drosophila 5C actin. Glycerol-d_8_ is almost completely depleted at ~ 30 h. Cells were grown an additional 8 h to ensure complete depletion of glycerol-d_8_ (see text)

To avoid medium exchange, we introduced three major changes to our original *P. pastoris* expression protocol: (1) optimization of BMGH medium to reduce glycerol concentration, (2) measurement of glycerol in the medium during the final growth stage to ensure complete depletion of glycerol before induction and (3) maintaining culture pH at 6.0 throughout the growth (Fig. 1). These conditions will support robust growth while limiting use of deuterated glycerol and affording minimal inhibition of AOX1, enabling induction to be performed in the final growth stage without requiring exchange of the medium. To find the optimal minimal concentration of glycerol, we grew *P. pastoris* harboring plasmids encoding drosophila 5C actin in BMGH with different glycerol concentrations (0.2, 0.4, 0.5, 0.6, 0.8 and 1%, w/v). In the final growth stage (after 32 h), actin was expressed by adding 0.5% (v/v) methanol. We found that 0.4–0.5% glycerol is optimal for actin expression (Fig. S1). We also observed that growth saturates at the same OD_600_ irrespective of glycerol concentrations beyond 0.4%. This indicates that other component(s)/condition(s) (probably YNB, biological oxygen demand, pH) become limiting in the medium (Heyland et al. 2011). Thus, we further optimized the medium composition by doubling the concentrations of YNB, biotin and histidine compared to the previous protocol. We note that YNB does not contain any carbon sources, so the large majority of carbon in the medium derives from glycerol. Table 1 shows the compositions of the optimized (BMGH*) and previously used BMGH media. The optimized medium contains 50% less deuterated glycerol, reducing the total cost by ~ 17–20% and facilitating faster depletion of glycerol prior to induction. Despite the lower glycerol concentration, the final cell density in the new protocol is essentially equal to that of the previous protocol (Fig. 2a), again suggesting that factors other than carbon are limiting for growth (Heyland et al. 2011).

In addition to minimizing use of deuterated glycerol in the final BMGH growth, we also optimized the time before induction to ensure complete depletion of glycerol to prevent inhibition of the AOX1 promoter. Using a commercially available kit, we measured glycerol in the medium throughout the final BMGH growth. As shown in Fig. 2b glycerol is almost completely consumed at ~ 30 h (OD_600_ ~ 20). In a typical procedure we allowed cells to grow another ~ 8 h beyond this point and then induced protein expression with 0.5% (v/v) d_4_-methanol, which also acts as a carbon source. If ^1^H/^13^C-labeling of isoleucine δ-methyl groups was desired, we also added methyl-^13^C, 3,3-d_2_ ketobutyric acid (100 mg/L) 3 h prior to induction. Thus, by monitoring glycerol levels we were able to induce directly in the depleted BMGH, eliminating the need to exchange the medium in the final large volume growth stage, reducing the ^2^H_2_O cost of the full protocol by nearly 50%. Note that once glycerol has been optimized, the timing of glycerol consumption and cell growth are quite reproducible, so that glycerol does not need to be directly monitored in subsequent cultures.

**Fig. 3:**
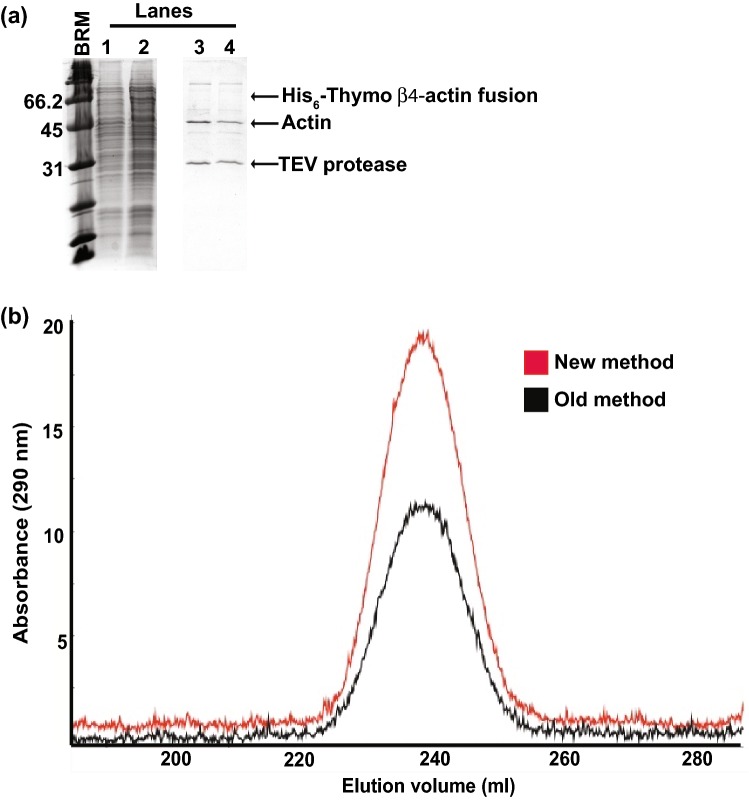
Comparison of overexpression of drosophila 5C actin purified from cultures grown using the old and new protocols. (**a**) SDS PAGE of overexpressed actin. Gel segments show molecular weight markers (BRM; Broad Range protein molecular weight Markers), initial cell pellets (lanes 1–2) and the final purified proteins (lanes 3–4) of the old (lanes 2, 4) and new (lanes 1, 3) protocols. Full gel showing all steps of purification is in Fig. S2. Arrows indicate locations of the expressed His_6_-thymosin β4-actin fusion, actin, and the TEV protease used to cleave off the His_6_-thymosin β4-actin tag. (**b**) Superdex 200 gel filtration chromatography trace of actin purified from cultures grown in old (black) and new (red) media. Proteins eluted at identical volumes

In a third aspect of optimization, we maintained the pH of the medium at 6.0 throughout the final growth stage culture, before and after induction. While *P. pastoris* can grow over a wide pH range, from 3 to 7, the growth rate is optimal at pH 6 (Eissazadeh et al. 2017). Without adjustment, the pH drops steadily as cell density in the culture increases due to the production of metabolic by-products. In an unregulated actin preparation, the pH drops to < 4.5 by the time of cell harvest. To ensure maximal yeast growth rate, we adjusted the pH of the initial BMGH medium to pH 6.0 using 10 M KOH. This produced a higher grown rate, and ultimately higher protein expression, than in cultures where pH was not adjusted (Figs. 2a, 3a).

Together, these optimization procedures result in an 50-60% decrease in glycerol-d_8_ (10 g vs 4–5 g per liter of final culture) and 50% decrease in total ^2^H_2_O (2000 mL vs 1000 mL per liter of final culture). Given current prices of these reagents, the optimized protocol is approximately 50% less expensive than our previously reported procedures (Clark et al. 2015).

### Actin expression is increased by ~ 30% using the optimized protocol

To compare the yield and quality of protein generated by the previous and new methods, we used them in parallel to express drosophila 5C actin. Figures 3a shows SDS PAGE gels obtained during purification of the two actin samples. It is evident that actin expression is higher on a culture volume basis using the new method. Proteins expressed using both methods showed identical gel filtration profiles in the final step of purification (Fig. 3b), suggesting similar quality of the materials. After purification an actin yield of 4 mg per liter of culture was obtained using the optimized protocol, while the old method produced only 3 mg per liter. Thus, the new protocol yields an approximately 30% increase in yield. Given the ~ 50% decrease in cost per liter of culture, this increase in yield produces a ~ 65% decrease in cost per final mass of overexpressed protein.

### Isotope enrichment is not compromised in the new method

**Fig. 4:**
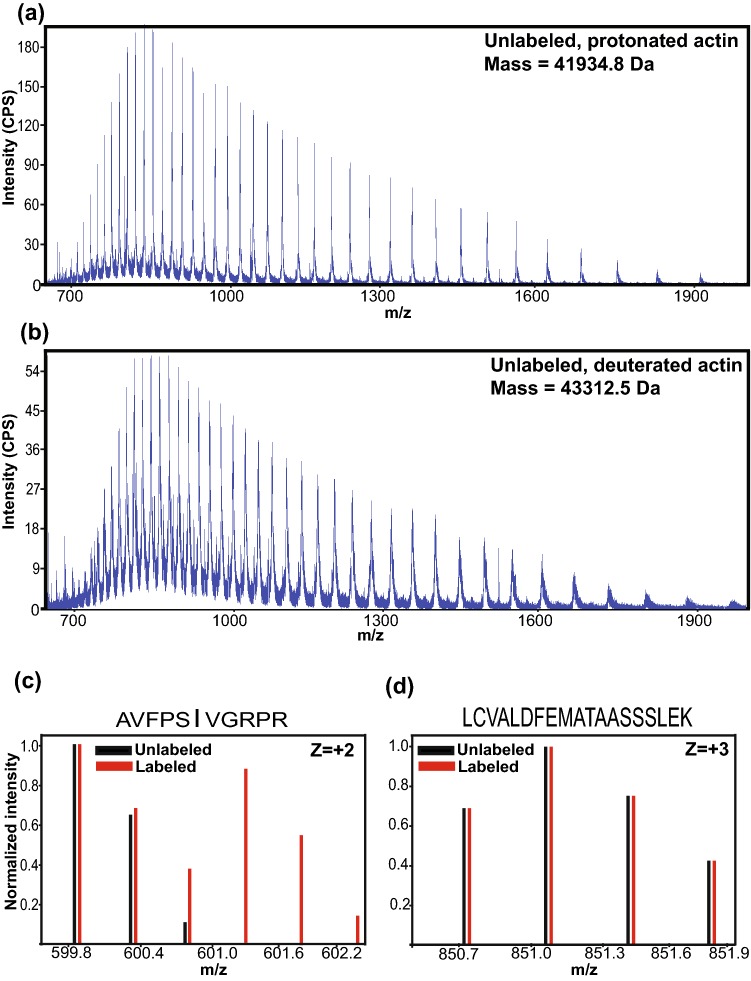
Determination of isotope incorporation efficiency. ESI-LC/MS spectra of protonated (**a**) and deuterated actin (**b**). Comparison of mass determined from the spectra shown in (**a**) and (**b**) indicates an overall 92% deuterium labeling at non-exchangeable sites. (**c**) and (**d**) Mass spectra showing isotopic distribution of tryptic peptides containing an isoleucine (**c**) or lacking an isoleucine (**d**) from actin labeled at isoleucine with ^1^H_3_^13^C at δ1 and ^2^H_2_^12^C at γ1 and otherwise fully protonated (red bars, “labeled”) or simply fully protonated (black bars, “unlabeled”)

To determine the overall deuteration level produced by the new protocol, we used ESI-LC/MS to measure the intact mass of actin samples prepared from either protonated or deuterated media, without ^13^C isoleucine labeling in both cases (Fig. 4a, b). The difference in mass between these samples, corrected for back-exchange of protons to exchangeable sites during processing for mass spectrometry, shows an overall deuteration level of 92% in the latter. This value is highly similar to the ~ 90% deuteration reported previously using the old protocol (Clark et al. 2015).

To quantitatively determine the efficiency of isoleucine labeling, we performed tryptic digestion on actin samples prepared from yeast grown in protonated media with or without the isoleucine precursor [4-^13^C-3,3- ^2^H_2_] α-ketobutyric acid, followed by mass spectrometry. Incorporation of this precursor leads to an increase in mass of 3 Da for each isoleucine. Comparison of the isotopic distributions of peptides from labeled and unlabeled samples was used to quantify the isotopic enrichment at isoleucine (see “Materials and methods”). Figures 4c and d show the isotopic distribution of tryptic fragments from unlabeled (black) and labeled (red) samples for peptides that contain (Fig. 4c) or lack (Fig. 4d) isoleucine. Isoleucine-containing peptides from the labeled protein show an increase in intensity in the M + 3 species relative to the same peptide from the unlabeled protein (Fig. 4c). Peptides that do not contain isoleucine have similar isotope distributions in both the labeled and unlabeled samples (Fig. 4d). Analysis of the mass spectrometry data on four isoleucine-containing peptides with strong signals revealed an average label enrichment at isoleucines of 46.6 ± 1.4% (see “Materials and methods”), again very similar to that achieved previously (50%) using the old protocol (Clark et al. 2015).

**Fig. 5:**
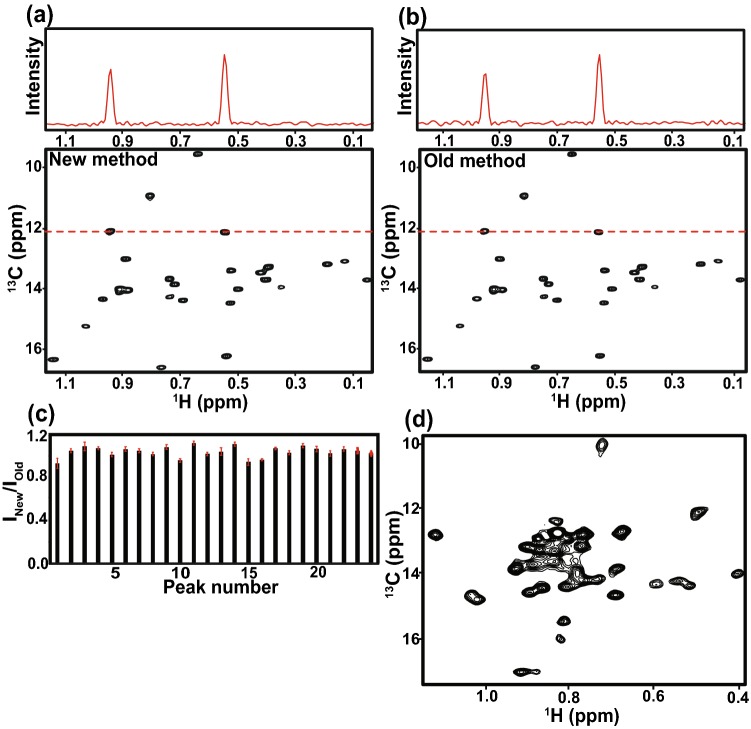
^1^H/^13^C methyl TROSY HMQC spectra and comparison of isotope enrichment in proteins overexpressed in *P. pastoris*. (**a**) and (**b**) ^1^H/^13^C methyl TROSY HMQC spectra acquired on ^1^H/^13^C isoleucine δ-methyl-labeled drosophila 5C actin (22.5 uM each) prepared using old and new protocols. A horizontal slice extracted from the 2D spectrum at 11.9 ppm in the ^13^C dimension is shown in the top panel. (**c**) S/N ratio of intensities for well resolved peaks in ^1^H/^13^C methyl TROSY HMQC spectra of actin purified using the old and new protocols. Error bars represent errors propagated from the measurement of noise in the NMR spectra. (**d**) ^1^H/^13^C methyl TROSY HMQC spectrum of ^1^H/^13^C isoleucine δ-methyl-labeled A_2A_ receptor (see text for details)

To further assess isotope enrichment produced by the two protocols, we measured the intensity of well-resolved resonances in ^1^H/^13^C HMQC TROSY spectra of ^1^H/^13^C isoleucine δ-methyl-labeled actin generated by each of them (Fig. 5). Both spectra were recorded on samples of 22.5 µM protein concentration with identical buffer conditions, acquisition parameters and temperature. Actin is a 42 kDa protein with 27 isoleucine residues, and both spectra show 27 peaks. Figure 5c shows the S/N ratio of spectra resulting from the two expression protocols, organized by ^13^C chemical shift. Within experimental error the peak intensities are identical, strongly suggesting that isotope enrichment is highly similar in proteins prepared by the two methods (Fig. 5c).

### The new method is also effective for a recombinant eukaryotic integral membrane protein

To further explore the generality of the new protocol, we used it to generate samples of the adenosine A_2A_ receptor, ADORA2A, an integral membrane protein and G-protein coupled receptor (GPCR). The new protocol produced equivalent yields of ADORA2A per liter of culture (~ 1 mg/L) as before (Clark et al. 2017), but at ~ 50% reduced cost. Figure 5d shows a ^1^H/^13^C methyl TROSY spectrum of ~ 100 µM deuterated, ^1^H/^13^C isoleucine δ-methyl-labeled A_2A_ receptor in 0.05% w/w n-Dodecyl-d_25_-β-d-maltopyranoside (Anatrace). The spectrum is of high quality, and 25 resonances can be observed for the 29 isoleucines in the protein.

## Conclusion

Methyl-containing amino acids (Ala, Leu, Val, Ile, Thr, Met) represent ~ 35% of the amino acids in soluble proteins, and up to 45% of α-helical membrane proteins (Kurauskas et al. 2017). Methyl groups have favorable NMR relaxation properties, which results in sharp signals in ^1^H-^13^C spectra (Rosenzweig and Kay 2014). Thus, methyl groups are ideal probes for NMR analyses of proteins. Recently, methyl TROSY spectra of deuterated, ^1^H/^13^C methyl labeled proteins have been used to study the structure and dynamics of large assemblies in the 100 kDa–1 MDa range (Clark et al. 2017; Ruschak and Kay 2012). One limitation to such approaches is the necessity of recombinantly expressing certain eukaryotic proteins in yeast, which can be quite expensive. Here we have described an efficient and robust method for expressing deuterated, ^1^H/^13^C isoleucine δ-methyl-labeled proteins in the yeast *P. pastoris*. Compared to our previous protocol, this method reduces the cost of labeling per liter of cell culture by ≥ 50% without compromising isotope enrichment. We have successfully implemented this method to label the eukaryotic proteins actin and the adenosine A_2A_ receptor, an integral membrane protein. It seems likely that costs could be further decreased by using a small scale fermenter where growth conditions could be most optimally and precisely regulated. We note that our protocol is optimized for expression of proteins in baffled Erlenmeyer flasks, and a different set of optimizations might be required for protein expression in fermenters.

We anticipate that it should be possible to extend the procedures here to label other methyl groups in proteins expressed in *P pastoris*. Recently, ^1^H/^13^C methyl labeling of leucine and valine in a deuterated background has been achieved in *P. pastoris* by lowering the pH of the induction medium (Suzuki et al. 2018), a modification that should be compatible with the key glycerol manipulations employed here. In addition, our preliminary data suggest that it should be possible to ^1^H/^13^C methyl label methionine residues in yeast analogously (data not shown). Methionine is a particularly valuable probe, given that it has a long and flexible side chain with a highly polarizable sulfur atom, features that facilitate many protein–protein interactions (Wiesner and Sprangers 2015). The availability of cost-effective methods to generate fully deuterated eukaryotic proteins with ^1^H/^13^C labeling at isoleucine/valine/leucine/methionine methyl groups by overexpression in yeast will significantly expand the scope of methyl TROSY NMR methods.

## Electronic supplementary material

Below is the link to the electronic supplementary material.

Supplementary material 1 (PDF 2486 kb)
