# Supplementary material for: Improved strategy for isoleucine 1H/13C methyl labeling in Pichia pastoris
Source: J Biomol NMR. 2019 Sep 20;73(12):687–97. doi: 10.1007/s10858-019-00281-1 (PMC6875547; doi:10.1007/s10858-019-00281-1)
Supplement: Supplementary file 1 — Supplementary material 1 (PDF 2486 kb) [file 10858_2019_281_MOESM1_ESM.pdf]

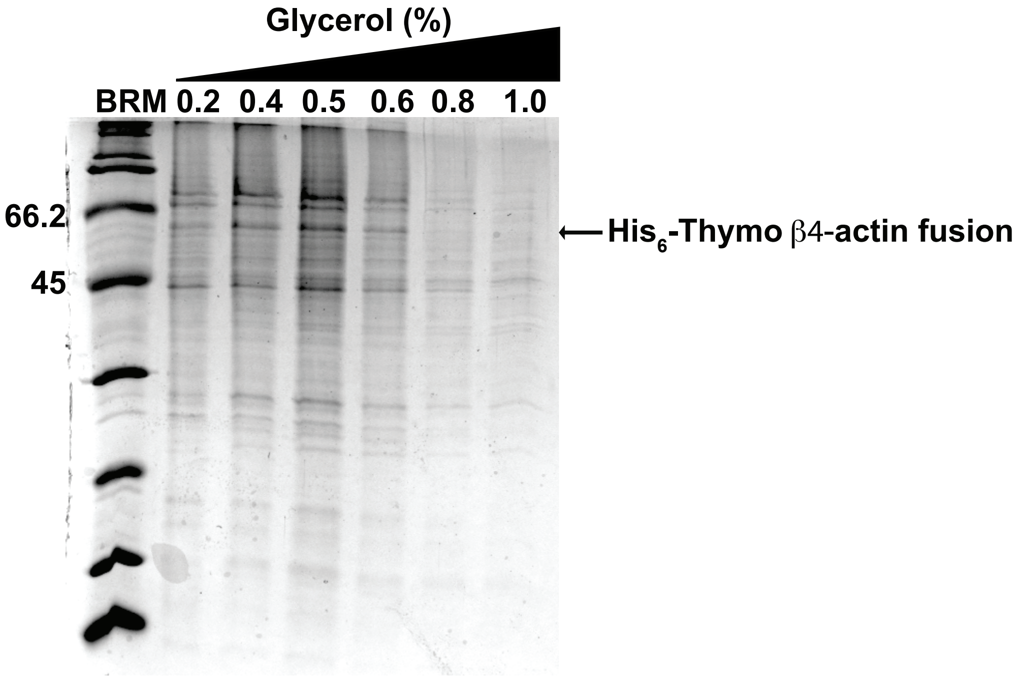

BRM = Broad range Marker

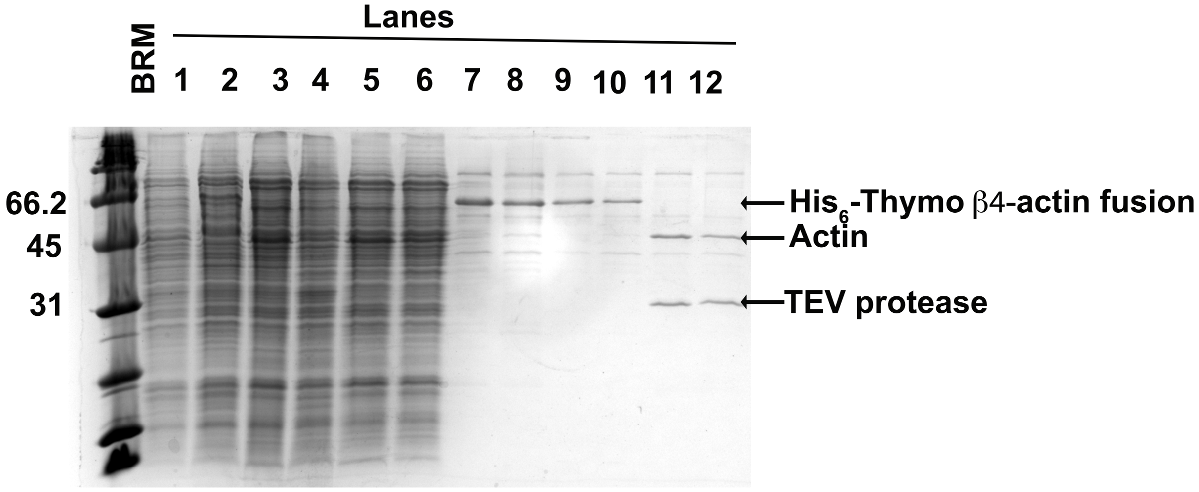

BRM = Broad range marker

1 = Pellet (New)

2 = Pellet (Old)

3 = Clear lysate (New)

4 = Clear lysate (old)

5 = Flow through (New)

6 = Flow through (Old)

7 = Elution I (New)

8 = Elution I (Old)

9 = Elution II (New)

10 = Elution II (Old)

11 = +TEV (New)

12 = +TEV (Old)

Supplementary Materials for

Improved strategy for isoleucine $^{1}H/^{13}C$ methyl labelling in *Pichia pastoris*

Rustam Ali[1,2*], Lindsay D. Clark[1,3], Jacob A. Zahm[1,2,4], Andrew Lemoff[5], Karthik Ramesh[1], Daniel M. Rosenbaum[1] and Michael K. Rosen[1,2*]

[1]Department of Biophysics, University of Texas Southwestern Medical Center, Dallas, TX, 75390, USA

[2]Howard Hughes Medical Institute, University of Texas Southwestern Medical Center, Dallas, TX, 75390, USA

[3]Current address: Department of Cell Biology, Harvard Medical School, 240 Longwood Avenue Boston, MA, 02115

[4]Current address: Department of Biochemistry and Molecular Pharmacology, 130 Longwood Avenue
Boston, MA, 02115

[5]Department of Biochemistry, University of Texas Southwestern Medical Center, Dallas, TX, 75390, USA

Purification of deuterated actin

Cells harvested from 1 liter of deuterated actin culture were suspended in ~50 ml lysis buffer containing 50 mM TRIS (pH 8.0), 10 mM imidazole (pH 7.5), 300 mM KCl, 0.2 mM ATP, 0.1 mM $CaCl_2$, 1 mM PMSF 1 mM β–mercaptoethanol (BME**)**, Leupeptin (1 μg/ml), 1 mM Benzamidine and Antipain (1 μg/ml). Resuspended cells were lysed by four passes through a microfluidizer (Microfluidics M-110P) at 25,000 psi. Cell debris was removed by centrifugation at 47807×g for 45 minutes. The resulting supernatant was loaded on 5 ml of Ni-NTA (QIAGEN) slurry, equilibrated with the lysis buffer followed by extensive washes (10 CV) with buffers WBI (20 mM Tris pH 8, 10 mM imidazole, 1 mM β-mercaptoethanol, 500 mM KCl, 0.2 mM ATP, 0.1 mM $CaCl_2$) and WBII (20 mM Tris pH 8, 20 mM imidazole, 1 mM β-mercaptoethanol, 100 mM KCl, 0.2 mM ATP, 0.1 mM $CaCl_2$). $(His)_6$-thymosin β4 actin was eluted in two 25 ml fractions of WBII buffer containing 500 mM imidazole at pH 7.5. The $(His)_6$-thymosin β4 tag, was removed by treatment with TEV protease overnight at 4 °C. The reaction mixture was diluted with ion exchange buffer A (10 mM imidazole, pH 7, 0.1 mM $CaCl_2$, 0.2 mM ATP, 50 mM NaCl, 1 mM β-mercaptoethanol) and loaded on a source15Q anion exchange column (GE Healthcare) equilibrated with buffer A. Actin was eluted with a linear gradient of buffer B (buffer A containing 1 M NaCl). Actin containing fractions were pooled and dialyzed against buffer B, and loaded onto 5 ml of Ni-NTA beads to remove residual TEV and $(His)_6$-thymosin β4 tag. The column flow through was loaded on an SD200 column (GE Healthcare, 320 ml) equilibrated with 10 mM HEPES, pH 7, 50 mM KCl, 0.2 mM $CaCl_2$, 0.2 mM ATP, 1 mM TCEP, 1 mM NaN_3. Actin fractions were pooled, concentrated and dialyzed in NMR buffer (10 mM HEPES pH 7, 50 mM KCl, 0.1 mM $CaCl_2$, 0.2 mM ATP, 1 mM TCEP, 1 mM NaN_3 in 100% D_2O.)

Glycerol optimization in BMGH medium:

To find the optimal glycerol concentration for protein expression in BMGH medium, we grew *P. pastoris* harboring plasmids encoding drosophila 5C actin in 10 ml of BMGH with different glycerol concentrations (0.2, 0.4, 0.5, 0.6, 0.8 and 1%, w/v). In the final growth stage (after 32 hours), actin was expressed by adding 0.5% methanol and allowed to grow for an additional 48 hours. Cells were harvested by centrifuging at 4700 g for 30 minutes. Media was decanted and each cell pellet was resuspended in 2.5 ml of lysis buffer containing 50 mM Tris (pH

8.0), 10 mM imidazole (pH 7.5), 300 mM KCl, 0.2 mM ATP, 0.1 mM $CaCl_2$, 1 mM PMSF 1 mM β−mercaptoethanol, Leupeptin (1 μg/ml), 1 mM Benzamidine and Antipain (1 μg/ml). An equal volume of glass beads (500 μm diameter) was added to each resuspension and vortexed vigorously for 3 x 5 minutes with an interval of 5 minutes between each cycle. The lysates were clarified by centrifuging at 48000×g for 30 minutes. Each supernatant was loaded separately on 250 μl of pre-equilibrated Ni-NTA beads, washed and eluted in 250 μl. The buffer composition for these Ni-NTA purifications was identical to that described above for actin. A 12 μl aliquot of each eluate was loaded on a 15% SDS PAGE to analyze protein expression (Figure S1).

Purification of deuterated adenosine A2A receptor

Frozen cell pellets were thawed and resuspended in lysis buffer (PBS containing 10% glycerol, 4 mM theophylline, 2 mM EDTA, and protease inhibitors (160 μg/ml benzamidine, 2.5 μ/ml leupeptin, 1 mM PMSF, 1 uM E-64)). Cells were passed through a chilled high-pressure microfluidizer (Microfluidics M-110P) three to four times at 24,000 psi with a cooling period between passes. Zymolyase-20T powder (AMS Bio) was added to the total lysate at a concentration of 60 U/mL (3 mg/mL) and stirred at 37 °C for 1 hr. Total membranes were isolated by centrifugation at 140,000 g for 30 min. Membranes were washed by loose douncing in an equal volume of lysis buffer followed by an additional centrifugation step. Membranes were resuspended in hypotonic buffer (10 mM HEPES pH 7.5, 2 mM EDTA, 4 mM theophylline, protease inhibitors) and loosely dounced, followed by incubation and stirring at 4 °C for 30 min. Membranes were again centrifuged at 140,000 g for 30 min. To solubilize the receptor, membranes were dounced in buffer containing 500 mM NaCl, 50 mM HEPES pH 7.5, 20% glycerol, 1% DDM (Anatrace), 4 mM theophylline, and protease inhibitors and stirred for 2 hours at 4 °C. Insoluble material was removed by centrifugation at 140,000 g for 30 min. The clarified supernatant was incubated with loose TALON resin (Clontech) pre-equilibrated in 250 mM NaCl, 50 mM HEPES 7.5, 0.05% protonated DDM, 4 mM theophylline, and 30 mM imidazole. Imidazole was added to the supernatant to a final concentration of 30 mM and batch binding continued overnight at 4° C.

Following batch binding, the resin was packed into a gravity column and washed with 10 CV of wash/exchange buffer to exchange both the ligand and detergent (250 mM NaCl, 50 mM HEPES 7.5, 0.05% detuerated DDM, 30 μM ZM 241385 (Tocris), and 30 mM imidazole, prepared

in 100% D_2O). A_{2A} was eluted with exchange buffer supplemented with 300 mM imidazole, and then concentrated in 100 kD MWCO concentrators and injected on a Superdex200 column (GE Healthcare) equilibrated in 150 mM NaCl, 20 mM HEPES pH 7.5, 0.05% deuterated DDM, and 10 μM ZM241385 made in 100% D_2O. Pure protein fractions were concentrated to 250 μL for NMR experiments.

Supplementary Figure Legends

Figure S1. SDS PAGE gel showing His_6-thymosin β4-actin expression in BMGH media with different glycerol concentrations.

Figure S2. SDS PAGE gel showing intermediates in purification of deuterated actin. Lanes 1, 3, 5, 7, 9, 11 show cell pellet, cleared lysate, flow through of the Ni-NTA column, first and second elutions of $(His)_6$-thymosin β4 actin, and the TEV cleavage reaction, respectively, for the new expression protocol. Equivalent materials for the old expression protocol are shown in lanes 2, 4, 6, 8, 10, 12. The left-most, unnumbered lane shows broad range molecular weight markers, with relevant masses indicated. Arrows indicate locations of the expressed His_6-thymosin β4-actin fusion, actin, and the TEV protease used to cleave off the His_6-thymosin β4-actin tag.
